# Phase 2 study of glucarpidase in patients with delayed methotrexate elimination after high-dose methotrexate therapy

**DOI:** 10.1007/s00280-024-04664-6

**Published:** 2024-03-13

**Authors:** Atsushi Ogawa, Hiroshi Kawamoto, Junichi Hara, Atsushi Kikuta, Chitose Ogawa, Hiroaki Hiraga, Kenichi Yoshimura, Kazunari Miyairi, Reiko Omori, Tokihiro Ro, Yuna Kamei, Toshimi Kimura

**Affiliations:** 1 Department of Pediatrics, Niigata Cancer Center Hospital, 2-15-3 Kawagishi-cho Chuo-ku, Niigata, 951-8566 Japan; 2 Department of Pediatric and Allergy, Fujimi Clinic, Tokyo, Japan; 3 Department of Pediatrics Hematology/Oncology, Osaka City General Hospital, Osaka, Japan; 4 Department of Pediatric Oncology, Fukushima Medical University Hospital, Fukushima, Japan; 5 Department of Pediatric Oncology, National Cancer Center Hospital, Tokyo, Japan; 6 Department of Musculoskeletal Oncology, NHO Hokkaido Cancer Center, Sapporo, Japan; 7 Department of Biostatistics and Data Science, Graduate School of Medical Science Nagoya City University, Nagoya, Japan; 8 Ohara Pharmaceutical Co., Ltd., Tokyo, Japan; 9 Department of Pharmacy, Juntendo University Hospital, Tokyo, Japan

**Keywords:** HD-MTX, CPG2, Glucarpidase, Clinical study, Japan

## Abstract

**Purpose:**

High-dose methotrexate therapy (HD-MTX) is a standard treatment for various malignant tumors, but approximately 1–10% of patients experience delayed MTX elimination (DME) that can induce organ damage. Glucarpidase can hydrolyze MTX and thereby lower the level of active MTX in the blood. A multicenter, open-label, phase II investigator-initiated trial (CPG2-PII study) was conducted to evaluate glucarpidase rescue therapy in Japanese patients who showed DME after HD-MTX treatment. To confirm the robustness of this therapy, further corporate-sponsored clinical trial (OP-07-001 study) was conducted.

**Methods:**

The primary endpoint in the CPG2-PII study was to evaluate the proportion of patients of the percentage clinical important reduction (CIR) as an indicator of MTX concentration, which can be managed with leucovorin and supportive care. The primary endpoint of the OP-07-001 study was to evaluate the decreasing rate of plasma MTX concentration at 20 min after glucarpidase administration from the baseline for four patients. Glucarpidase was administered at a dose of 50 U/kg for 15 and 4 patients, respectively in the two studies, and safety was analyzed for each of them.

**Results:**

The rate of CIR was 76.9% (95% confidence interval, 46.2–95.0%) in the CPG2-PII study. The median reduction rate of plasma MTX was 98.83% in the OP-07-001 study. Hypersensitivity, blood bilirubin increased, and headache for each patient were the only study drug-related events.

**Conclusion:**

Glucarpidase showed an effect of reducing plasma MTX concentration in Japanese patients with DME as that observed in a previous US study, confirming its favorable safety and tolerability.

**Supplementary Information:**

The online version contains supplementary material available at 10.1007/s00280-024-04664-6.

## Introduction

High-dose methotrexate (MTX) therapy (HD-MTX) is the standard treatment for several malignant tumors including acute lymphatic leukemia, malignant lymphoma, and osteosarcoma [1–6]. The risk of HD-MTX therapy depends on the administration method, age, and physical condition. Delayed MTX clearance occurs in approximately 1–10% of patients, and can cause acute kidney injury (AKI), hepatotoxicity, mucositis, and cytopenias [7–15]. In such cases, supportive therapies for HD-MTX toxicity, such as vigorous hydration to prevent AKI due to MTX-related calculus and direct urothelial toxicity, urinary alkalinization with NaHCO_3_, and leucovorin (LV) rescue therapy, can be continued or increased, and in extreme cases dialysis may be required [16, 17]. However, with these measures it can take considerable time to lower the concentration of MTX in the blood to nontoxic levels [18, 19]. According to pooled data from 3,887 patients with osteosarcoma in 2004, grade 2 or higher renal impairment (WHO criteria) was observed in 68 patients (1.8%) despite these supportive therapies, and the treatment-related mortality remained high at 4.4% in such cases [15, 20]. MTX is primarily excreted by the renal route and may directly cause renal impairment and nephrotoxicity, which can cause delayed MTX elimination [21]. Therefore, alternative methods to manage patients with persistently elevated MTX concentrations for long periods are needed.

Glucarpidase (carboxypeptidase G2, CPG2) is a dimeric enzyme with a molecular weight of approximately 41,440 Da that rapidly hydrolyzes MTX to its inactive metabolites, 4-deoxy-4-amino-N^10^-methylpteroic acid (DAMPA) and glutamate [22, 23]. The direct metabolism of MTX is necessary as renal elimination is impaired in patients with delayed MTX clearance. Glucarpidase has become a semi-standard treatment in Europe and the United States as an effective method to eliminate extracellular MTX with an aim of reducing toxicity of delayed MTX elimination without inhibiting the antitumor effect of MTX [1–6]. However, in Japan, increasing the LV dose and performing dialysis as necessary are the only treatments currently recommended for delayed MTX clearance, and glucarpidase has not been used. Although most patients with delayed MTX clearance can recover from mucositis or bacteremia, increasing the LV dose may affect the treatment plan for the original malignant tumor, increasing recurrence and decreasing the response rate [24, 25]. Therefore, the clinical application of glucarpidase is required in Japan.

To ensure glucarpidase was available for patient use before the launch of the product, an investigator-initiated study was conducted using product provided by Protherics Medicines Development Ltd.; glucarpidase was approved by the FDA in 2012 and by the EMA in 2022. For clinical development of glucarpidase in Japan, a phase I study (CPG2-PI study) was first conducted for healthy Japanese adults. This study included 16 patients that were randomized for administration of glucarpidase with eight patients each assigned to two cohorts at the dose levels of 20 U/kg (cohort 1) and 50 U/kg (cohort 2), with one repeated dose, respectively. Tolerability was indicated at both these doses [26], although adverse events were observed in 11 of 16 patients (68.8%) and the adverse events rates in cohorts 1 and 2 were 75.0% and 62.5%, respectively. No clinically relevant differences between glucarpidase dose levels were seen. None of the patients experienced dose-limiting toxicity, and no deaths, other serious adverse events, or other significant adverse events occurred [26]. Thus, a glucarpidase dose of 50 U/kg was used in the phase II study (CPG2-PII study) to evaluate the efficacy, safety and pharmacokinetic analysis. However, the efficacy result in this study was suggested to be overestimated due to issues in sample processing. Therefore, to improve the robustness of the findings from CPG2-PII, a subsequent study (OP-07-001) was conducted by Ohara Pharmaceutical Co., Ltd that enrolled four patients with high MTX concentrations. Herein, we report on these two studies to evaluate the decrease in MTX concentration following glucarpidase treatment and occurrence of any adverse events associated with this.

## Materials and methods

### Study design

CPG2-PII and OP-07-001 studies were conducted as phase II multicenter studies that investigated the efficacy, safety, and pharmacokinetics of glucarpidase in patients with delayed MTX clearance after HD-MTX therapy. glucarpidase has demonstrated high efficacy in the United States and Europe in compassionate use. Therefore, those trials were conducted without a comparative group because it is ethically problematic to include patients who are not treated with glucarpidase.

The CPG2-PII study, multicenter, open-label, phase 2 study was conducted to evaluate the potential of glucarpidase to reduce a high blood concentration of MTX in Japanese pediatric and adult patients due to delayed clearance of MTX (jRCT2091220097). Blood samples for measurement of plasma MTX concentration were centrifuged at 4 °C and cryopreserved at − 70 °C or lower. However, it was suggested that the effect of glucarpidase was overestimated because the enzymatic activity of glucarpidase may not be inactivated under these conditions. Therefore, study OP-07-001 (jRCT2080225030) was conducted to confirm the results in CPG2-PII. In OP-07-001, blood collection tubes containing EDTA and citric acid were used to inactivate glucarpidase, which is a zinc-dependent peptidolytic enzyme and functions optimally at a pH near 7.5; thus, the enzyme would be inactivated by chelating zinc and changing the pH to acidic conditions.

These studies were conducted in accordance with the principles of the World Medical Association Declaration of Helsinki, the Good Clinical Practice guidelines, and local regulations. The study protocol was approved by the institutional review board of each institution. Written informed consent was obtained from the patients for participation and enrolment to the clinical trial.

### Patients

In the CPG2-PII study, eligible patients were enrolled who had an abnormally high MTX concentration that considerably exceeded the safe concentration range at each time point from 22 to 70 h after starting administration of HD-MTX or those whose MTX concentration exceeded the safe concentration range and whose serum creatinine increased after starting HD-MTX administration. In the case of a patient who had not been treated with glucarpidase, any one of the criteria i) to vii) was met. The criteria were as follows: (i) Blood MTX levels of ≥ 50 µmol/L at least 22 h after the start of MTX administration, (ii) Blood MTX levels of ≥ 5 µmol/L at least 40 h after the start of MTX administration, (iii) Blood MTX levels of ≥ 2 µmol/L at least 46 h after the start of MTX administration, (iv) MTX blood concentration of ≥ 1 µmol/L and signs of acute renal failure at 40 h or later after the start of MTX, (v) MTX blood concentration of ≥ 0.4 µmol/L and signs of acute renal failure at 46 h or later after the start of MTX, (vi) MTX blood concentration of ≥ 0.1 µmol/L (with a dose of MTX 1–3.5 g/m^2^) at 70 h or later after the start of MTX, (vii) MTX blood concentration of ≥ 0.3 µmol/L (with a dose of MTX > 3.5 g/m^2^) at 70 h or later after the start of MTX. Additionally, in the patient who had been treated with glucarpidase showed signs of acute renal failure, and MTX blood concentrations of ≥ 50 µmol/L at 22 h or later after the start of MTX. Acute renal failure was defined as (i) the levels of serum creatinine exceeding the upper limits specified in the following table, at no less than 12 h after the start of MTX administration, or a creatinine clearance or glomerular filtration rate (calculated value or actual measurement, for both) of < 70 mL/min or (ii) levels of serum creatinine showing more than twofold increase from before MTX administration or showing more than 1.5-fold increase at the last two sequential blood samplings as well as continuing to increase. In the OP-07-001 study, eligible patients who showed an abnormally high MTX concentration that considerably exceeded the normal concentration range were enrolled at each time point from 22 to 70 h after the onset of HD-MTX administration. Patients who passed at least 15 h after the end of MTX treatment and met any of the following criteria, 1)–4): (1) the blood MTX concentration measured at the site is > 50 µmol/L at 22 h or more after the start of MTX administration. (2) the blood MTX concentration measured at the site is > 5 µmol/L at 40 h or more after MTX administration. (3) the blood MTX concentration measured at the site is > 2 µmol/L at 46 h or more after MTX administration. (4) the blood MTX concentration measured at the site is > 1 µmol/L at 40 h or more after the start of MTX treatment, a sign of AKI was observed. A sign of AKI was defined as meeting any of the following criteria, (i), (ii), and (iii). (i) Serum creatinine level after the MTX administration was higher than upper limit of the site reference value. (ii) At least 1.5-fold increase in the serum creatinine level from baseline after MTX administration. (iii) Increase in the serum creatinine levels by 0.3 mg/dL or higher within 48 h. The inclusion criteria in each study are provided in Supplementary Table S1.

Patients were excluded if they needed any concomitant treatment with drugs that affect MTX clearance/metabolism (e.g., penicillins, cephalosporins, aminoglycosides, tetracycline, nonsteroidal anti-inflammatory drugs, loop diuretics, thiazides, and probenecid) after high-dose MTX therapy and any episode of hypersensitivity reaction for glucarpidase or its additives (lactose, Tris-HCl buffer solution). No patients who were receiving prohibited concomitant medicines at the enrolment.

### Treatments

The safety of administering two doses of 50 U/kg per dose at an interval of 48 h was confirmed in the CPG2-PI study. The dose of glucarpidase that had been used for compassionate use in Europe and the United States had been 50 U/kg in all studies. The pharmacokinetics (PK) and drug interaction with LV studies performed in the United States [27] were also conducted at a dose of 50 U/kg. Taking these data into consideration, 50 U/kg was used subsequently in this trial. The dose of glucarpidase was calculated by rounding off the patient body weight to the nearest integer and multiplying this by 50. The glucarpidase 1000 U/vial was reconstituted with 1 mL of sterile saline for injection to adjust the dose. Patients with a MTX concentration ≥ 1 µmol/L, according to local laboratory results at 48 h after the first dose, could receive a second dose of glucarpidase dose at the same concentration as the initial dose in the CPG2-PII study. The same dose was used in the OP-07-001 study but glucarpidase was only administered once.

### Outcomes and assessments

In the CPG2-PII study, the primary endpoint was to evaluate the proportion of patients who achieved clinically important reduction (CIR), defined as < 1 µmol/L of the MTX plasma levels after the initial injection lasting for at least 5 days. The occurrence of adverse events or adverse drug reactions and the pharmacokinetic parameters of glucarpidase, MTX, DAMPA LV, and 5-MeTHF were evaluated as secondary endpoints. Renal impairment, pyrexia, neutropenic fever, mucositis, and infections and parasitosis, whose causality was related to MTX, were specified as MTX-related adverse events and their occurrence or nonoccurrence rate were evaluated. The observation period was defined as the interval between the start of observation and administration of glucarpidase, and the observation will end when the protocol treatment ended, and one of the following conditions (whichever comes first) was met: (i) It was assessed that the next course of administration of an antitumor drug can be started for the treatment of the primary disease. (ii) There were no adverse event of grade 2 (CTCAE) (excluding hair loss and abnormal test values). (iii) Adverse events of grade 3 that existed during the treatment period had all resolved to grade 2, and this state did not change for 3 days or longer. (iv) The patient died.

In OP-07-001, the decreasing rate of the plasma MTX concentration (central measurement) at 20 min after glucarpidase administration from the baseline measured immediately before administration was regarded as the primary endpoint. Secondary endpoints included the plasma MTX concentration and achievement of CIR, the occurrence of adverse events, adverse drug reactions, and pharmacokinetic parameters of glucarpidase. The CIR in OP-07-001 study was defined at all the scheduled blood sampling timepoints after the glucarpidase administration whether the plasma MTX concentration via the central measurement was below the threshold (1 µmol/L). The observation period was defined as the interval from enrolment day to 28 days after administration day of glucarpidase unless the patient met either (1) to (6) of the following criteria, (1) When it was judged that treatment for the target disease should be prioritized (chemotherapy was required regardless of the route of administration) when HD-MTX therapy was performed, (2) When the investigator or subinvestigator judges that the study should be discontinued because of occurrence of adverse events, (3) When the subject or his/her legally authorized representative requested to discontinue the study, (4) When the subject was found to be ineligible for the study, (5) When it was found impossible to perform the necessary observations/examinations because of the subject’s convenience or (6) When the investigator or subinvestigator judged that the study should be discontinued.

### Sample size

In the CPG2-PII study, considering that the number of patients with the target disease is rare, 18 patients were set to study the effectiveness and safety of the glucarpidase and the pharmacokinetic analysis of each analyte in a single dose, open-label design.

In the OP-07-001 study, in reference to the results of Study CPG2-PII, when the expected mean decreasing rate of the plasma MTX concentration (central measurement) was set to 99% and the standard deviation to 0.25, at least 3 patients were required to confirm that the mean decreasing rate exceeds 98.5% by one side t-test with a significance level set to 10% and power of 80%.

### Measurements

In the CPG2-PII study, blood samples for glucarpidase, MTX, and DAMPA analysis were collected on day 1 (before glucarpidase dosing, 20 min and 2 h after glucarpidase dosing, and before a second LV administration) and were collected on day 2 to 5 continuously. Blood samples for LV and 5-MeTHF analysis were collected on days 1 (2 and 3 h after glucarpidase dosing and before a second LV administration) and 2 (14–20 h after glucarpidase dosing, 1 h after LV dosing, and before next LV administration). glucarpidase levels were measured via immunoassay at a central testing laboratory. The concentrations of MTX, DAMPA, 5-MeTHF, and LV in plasma were measured using LC/MS/MS at the central laboratory while the MTX concentration was measured at a local laboratory, with an MTX assay kit. The sampling times are listed in Supplementary Table S2. Antibody tests for glucarpidase were performed on day 1 (before glucarpidase dosing) and 1, 3, and 6 months after glucarpidase dosing. Measurement of vital signs and body weight, and laboratory tests were performed.

In the OP-07-001 study, blood samples for glucarpidase analysis were collected on day 1 and 2 (before glucarpidase dosing and 20 min and 2, 24, and 48 h after glucarpidase dosing). Blood samples for MTX and DAMPA analysis were collected on day 1 to 5 (before glucarpidase dosing and 20 min and 2, 8, 24, 48, 72, and 96 h after glucarpidase dosing). glucarpidase was measured by immunoassay at the central laboratory, and MTX was measured by MTX assay kit at the local laboratory. MTX and DAMPA were measured via LC/MS/MS at the central laboratory. Vital signs and body weight were measured, and laboratory tests and 12-lead electrocardiogram were performed.

### Statistical analysis

In the CPG2-PII study, the primary endpoint was achievement of CIR that was the highest MTX concentration after administration of glucarpidase was below 1 µmol/L. A proportion of patients who achieved CIR was calculated as the primary analysis. A two-sided 95% confidence interval (CI) for the proportion of CIR was calculated using the exact method based on a binomial distribution. As secondary analysis, the number and proportion of patients with MTX-related adverse events, pharmacokinetic parameters of glucarpidase, MTX, DAMPA, LV, and 5-MeTHF, and incidence of the adverse events and adverse reactions were calculated.

In the OP-07-001 study, for the primary analysis, the decreasing rate of the plasma MTX concentration at 20 min after the start of glucarpidase administration from baseline and mean of the decreasing rate with one-sided 90% CI [lower limit] were calculated in the full analysis set. The datasets analyzed in this study are available from Ohara Pharmaceutical Co., Ltd.

## Results

The summary of the patient demographics in both studies are shown in Table 1. Individual data is shown in Supplementary Table S3. The CPG2-PII study was terminated before achieving the target sample size and only 15 patients were enrolled. The target diseases of HD-MTX therapy at the time of diagnosis were osteosarcoma in 9 patients (60.0%), which was the most common, followed by acute lymphocytic leukemia in 3 patients (20.0%), non-Hodgkin’s lymphoma in 2 patients (13.3%) and others (cerebellar medulloblastoma) in one patient (6.7%). Of the 15 patients, one patient who was registered twice and one patient whose plasma MTX concentration at the time of registration was < 1 µmol/L at the central laboratory were excluded from the primary analysis. Only one patient, the second registration case of the patient who registered twice was excluded from secondary analysis. Accordingly, 13 patients were included in the primary analysis and 14 in the secondary analysis. In the OP-07-001 study, 4 patients were enrolled and all were included in efficacy, safety, and pharmacokinetic analysis.

**Table 1:** Patient demographics

Characteristic	CPG2-PII		OP-07-001	
	*N* = 15	(%)	*N* = 4	(%)
Age:				
Median	15.0		9.5	
Range	1–75		2–79	
Gender:				
Female	6	40.0	1	25.0
Male	9	60.0	3	75.0
Diagnosis				
Acute lymphatic leukemia	3	20.0	3	75.0
Non-Hodgkin’s lymphoma	2	13.3	1	25.0
Osteosarcoma	9	60.0	0	0.0
Other	1	6.7	0	0.0
Body weight (kg)				
Median	47.0		33.85	
Range	10.7–78.1		12.6–62.2	
Blood MTX concentration* (µ mol/L)				
Median	51.0		2.60	
Range	1.02–692		2.28–3.10	

* At the time of diagnosis of delayed MTX clearance

### Efficacy result

In the CPG2-PII study, 10 patients (76.9%; 95% CI, 46.2–95.0%) achieved a CIR (two-tailed 95% CI) of the 13 patients included in the primary analysis. Nine (of 13) patients were administered a single dose and 8 of these achieved CIR (88.9%; two-tailed 95% CI, 51.8–99.7). Four (of 13) patients (Patient #1, 3, 8, and 15) were administered a second dose and 2 of these achieved CIR (50.0%; two-tailed 95% CI, 6.8–93.2). The MTX (local measurement)/plasma MTX (central measurement) concentrations at baseline of 3 patients who did not achieve CIR were 411/486, 21.8/22.8, and 51.0/26.9 µmol/L, respectively. For one patient (Patient #1), the MTX plasma concentration remained ≥ 1 µmol/L up to 48 h post-glucarpidase administration. However, this baseline of MTX plasma concentration for this patient exceeded 400 µmol/L and at 20 min after glucarpidase administration decreased to approximately 1% compared with the baseline. For 2 other patients (Patient #8 and #2), the plasma MTX concentration reduced to approximately 1% at 20 min after administration of glucarpidase and the MTX plasma concentration remained < 1 µmol/L up to 24 and 48 h. However, the plasma MTX concentration was ≥ 1 µmol/L at 48 and 72 h after administration of glucarpidase.

**Fig. 1:**
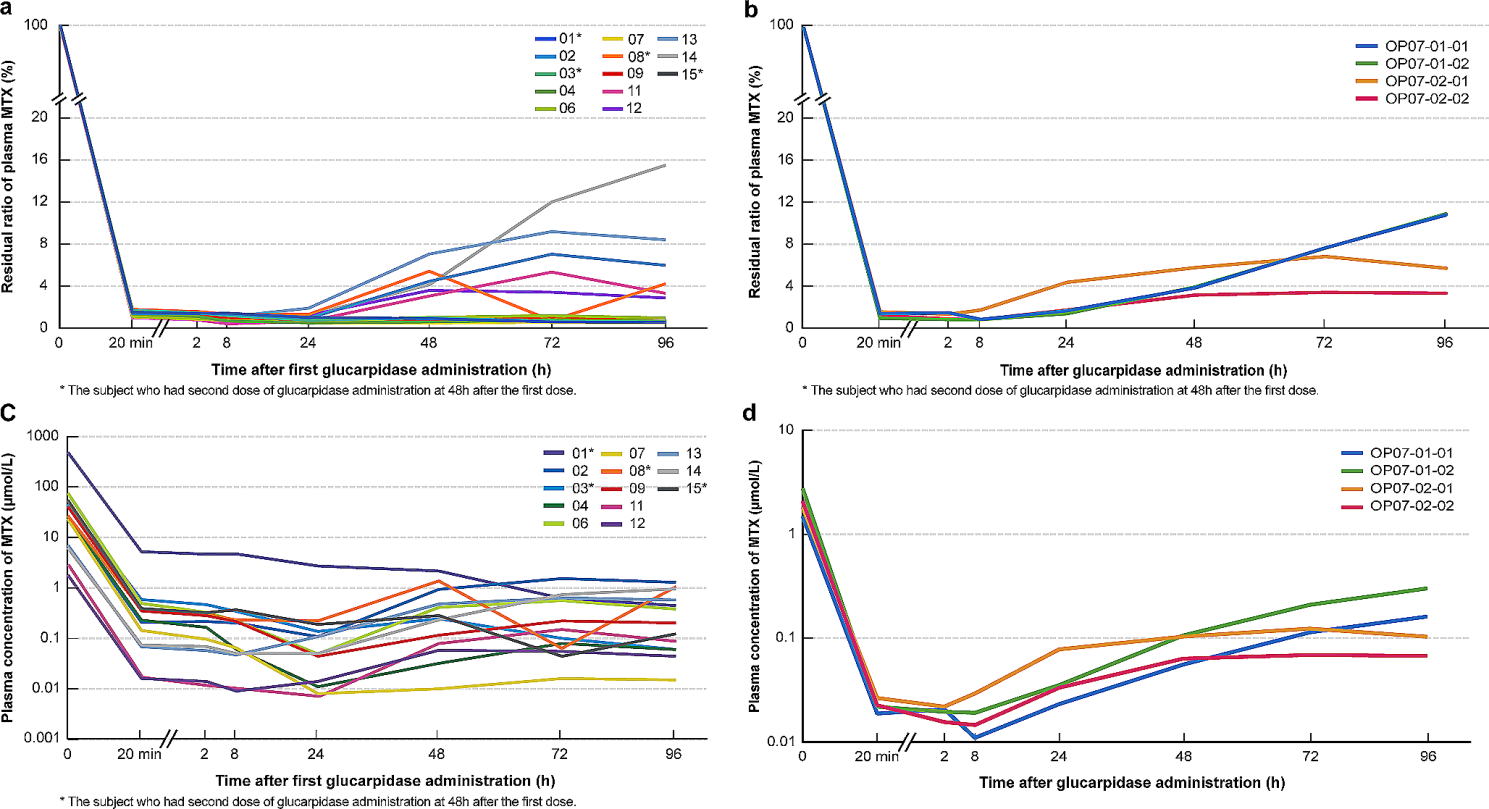
Time-course changes in the residual ration of plasma methotrexate (MTX) concentration in the CPG2-PII (**a**) and OP-07-001 (**b**) studies. Time-course changes in plasma MTX concentration for each patient in the CPG2-PII (**c**) and OP-07-001 (**d**) studies. The plasma MTX concentration 20 min after administration of glucarpidase (carboxypeptidase G2, CPG2) rapidly decreases compared with that before treatment with glucarpidase. Clinical important reduction (CIR) indicates the proportion of patients in whom the MTX after administration of glucarpidase does not exceed 1 µmol/L

**Fig. 2:**
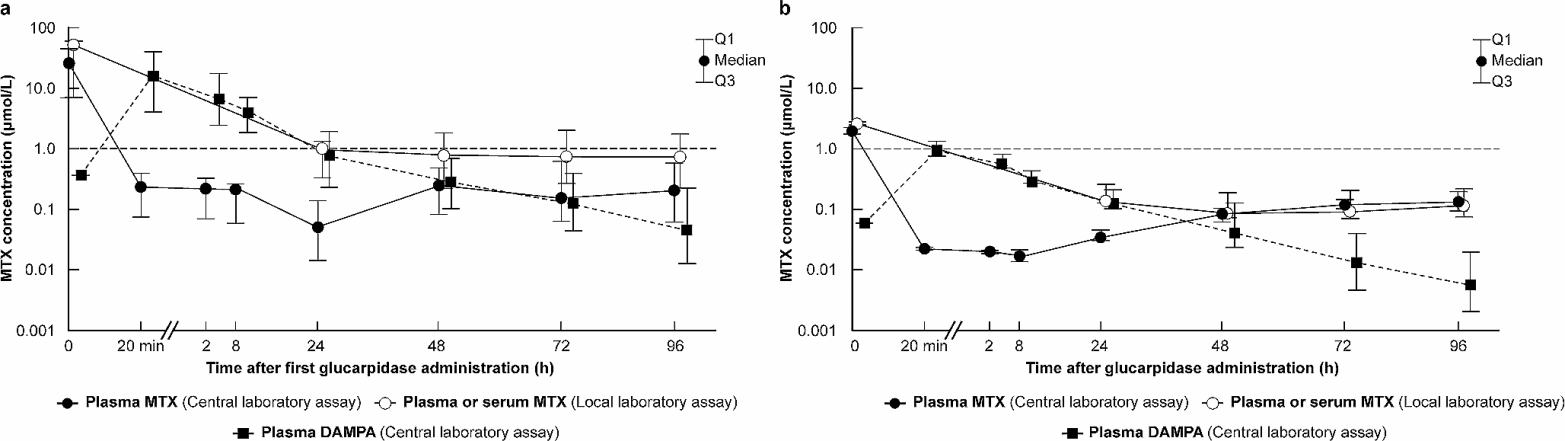
Changes in the methotrexate (MTX) concentration assayed at a local laboratory and in DAMPA and MTX assayed at a central laboratory in the CPG2-PII (**a**) and OP-07-001 (**b**) studies. The MTX concentration by local assay comprises MTX + DAMPA and differs from the plasma MTX concentration until approximately 48 h after administration of glucarpidase (carboxypeptidase G2, CPG2). Rebound occurs 48 h or later after administration of glucarpidase; however, the level of MTX in rebound is substantially lower than the original MTX level, and most do not exceed the 1-µmol/L threshold

In the OP-07-001 study, the CIR was achieved in all 4 patients (100.0%), with a median (minimum–maximum) estimated time to 1-µmol/L decreasing of 6.0 min (5–12 min). The MTX residual rate and MTX plasma concentration in both studies are shown in Fig. 1a, b, c and d. The mean decreasing rate of the plasma MTX concentration (central measurement) at 20 min after administration of glucarpidase was 99.1% and 98.9% in the CPG2-PII and OP-07-001 studies, respectively. The mean plasma concentration–time profiles of MTX measured at local laboratory and for DAMPA and MTX measured at the central laboratory are shown in Fig. 2a and b.

**Fig. 3:**
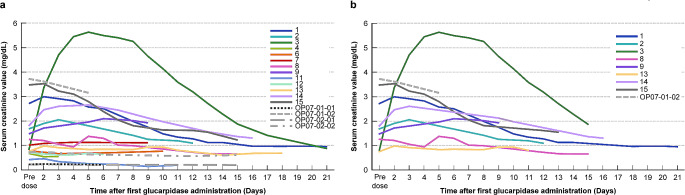
Time-course changes in the serum creatinine level in 17 patients in the primary analysis in both studies (**a**) and in patients who exceeded 1.5 times of the upper limit of the normal range in each institution at baseline or after administration of glucarpidase (carboxypeptidase G2, CPG2) (**b**). The serum creatinine levels increase until day 4 after administration and are maintained until day 8 after administration. Thereafter, the value returns to baseline on day 11 after administration and then decreases to below baseline by day 21 after administration. The baseline serum creatine levels of patients 1, 2, 8, 14, 15 and OP07-01-02 exceed 1.5 times of the upper limit of the normal range in each institution

Serum creatinine was used as an index of renal impairment and was measured from before administration of glucarpidase through to day 47 after administration. The level of serum creatinine increased until day 4 after administration and was maintained until day 8 after administration before returning to baseline on day 11 after administration and decreasing to below baseline by day 21 after administration. The serum creatinine results are shown in Fig. 3a and b for the 13 patients and 4 patients in primary analysis and those who exceeded the upper limit of the of normal range in each institution at baseline or after administration of glucarpidase in both studies, respectively. All 3 patients in CPG2-PII study who did not achieve CIR exceeded the upper limit of the normal range at baseline by 1.5-fold.

### Safety results

Table 2 lists all adverse events recorded for ≥ 20% of patients, all grade ≥ 3 adverse events and at the day of glucarpidase administration, regardless of relationship to glucarpidase in both studies. Regardless of adverse event grade, at least one occurred in all patients. In the CPG2-PII study, anaemia occurred most frequently (73.3%), followed by hypoalbuminaemia (60.0%), platelet count decreased (60.0%), neutrophil count decreased (53.3%), white blood cell count decreased (53.3%), and hyponatraemia (46.7%) in order of frequency. No adverse events were related to the glucarpidase activity. It was concluded that hypersensitivity (one case) and blood bilirubin increased levels were probably and possibly related to glucarpidase, respectively. The adverse events occurred on the day of glucarpidase administration for 6 patients (40.0%); however, only hypersensitivity was an adverse event related to glucarpidase. No deaths occurred in this study.

**Table 2:** Toxicities occurring in ≥ 20%, ≥Grade 3 or at the day of glucarpidase administration

System Organ Class Preferred Term	CPG2-PII (*N* = 15)						OP-07-001 (*N* = 4)					
	All Grades		≥Grade3		At the day of glucarpidase administration		All Grades		≥Grade3		At the day of glucarpidase administration	
	*N* of patients	(%)	*N* of patients	(%)	*N* of patients	(%)	*N* of patients	(%)	*N* of patients	(%)	*N* of patients	(%)
Any adverse event	15	100.0	13	86.7	6	40.0	4	100.0	4	100.0	2	50.0
Infections and infestations												
Enterocolitis infectious	1	6.7	1	6.7	0	0.0	0	0.0	0	0.0	0	0.0
Sepsis	1	6.7	1	6.7	0	0.0	0	0.0	0	0.0	0	0.0
Blood and lymphatic system disorders												
Anaemia	11	73.3	9	60.0	0	0.0	1	25.0	1	25.0	0	0.0
Febrile neutropenia	4	26.7	4	26.7	0	0.0	0	0.0	0	0.0	0	0.0
Metabolism and nutrition disorders												
Hypoalbuminaemia	9	60.0	0	0.0	0	0.0	0	0.0	0	0.0	0	0.0
Hyponatraemia	7	46.7	1	6.7	0	0.0	0	0.0	0	0.0	0	0.0
Hypokalaemia	6	40.0	4	26.7	0	0.0	1	25.0	1	25.0	0	0.0
Hypocalcaemia	5	33.3	2	13.3	1	6.7	0	0.0	0	0.0	0	0.0
Hypophosphataemia	4	26.7	0	0.0	0	0.0	0	0.0	0	0.0	0	0.0
Hyperuricaemia	0	0.0	0	0.0	0	0.0	1	25.0	0	0.0	0	0.0
Decreased appetite	2	13.3	0	0.0	1	6.7	0	0.0	0	0.0	0	0.0
Psychiatric disorders												
Insomnia	0	0.0	0	0.0	0	0.0	1	25.0	0	0.0	0	0.0
Nervous system disorders												
Headache	2	13.3	0	0.0	0	0.0	1	25.0	0	0.0	0	0.0
Peripheral motor neuropathy	1	6.7	1	6.7	1	6.7	0	0.0	0	0.0	0	0.0
Mental disorder												
Anxiety	1	6.7	0	0.0	1	6.7	0	0.0	0	0.0	0	0.0
Vascular disorders												
Hypertension	6	40.0	3	20.0	1	6.7	0	0.0	0	0.0	0	0.0
Gastrointestinal disorders												
Stomatitis	5	33.3	1	6.7	0	0.0	0	0.0	0	0.0	0	0.0
Vomiting	5	33.3	1	6.7	0	0.0	0	0.0	0	0.0	0	0.0
Diarrhoea	4	26.7	1	6.7	0	0.0	1	25.0	0	0.0	0	0.0
Constipation	3	20.0	0	0.0	1	6.7	0	0.0	0	0.0	0	0.0
Nausea	3	20.0	1	6.7	1	6.7	0	0.0	0	0.0	0	0.0
Cheilitis	0	0.0	0	0.0	0	0.0	1	25.0	0	0.0	0	0.0
Oral disorder	0	0.0	0	0.0	0	0.0	1	25.0	0	0.0	0	0.0
Abdominal pain	2	13.3	0	0.0	1	6.7	0	0.0	0	0.0	0	0.0
Renal and urinary disorders												
Acute kidney injury	1	6.7	1	6.7	0	0.0	0	0.0	0	0.0	0	0.0
Respiratory, thoracic and mediastinal disorders												
Hiccups	2	13.3	0	0.0	1	6.7	0	0.0	0	0.0	0	0.0
General disorders and administration site conditions												
Pyrexia	4	26.7	0	0.0	0	0.0	2	50.0	0	0.0	2	50.0
Mucosal disorder	1	6.7	1	6.7	0	0.0	0	0.0	0	0.0	0	0.0
Immune system disorder												
Hypersensitivity	1	6.7	0	0.0	1	6.7	0	0.0	0	0.0	0	0.0
Investigations												
Platelet count decreased	9	60.0	6	40.0	0	0.0	1	25.0	0	0.0	0	0.0
Neutrophil count decreased	8	53.3	6	40.0	0	0.0	2	50.0	2	50.0	0	0.0
White blood cell count decreased	8	53.3	5	33.3	0	0.0	2	50.0	1	25.0	0	0.0
Blood creatinine increased	5	33.3	1	6.7	0	0.0	0	0.0	0	0.0	0	0.0
Weight decreased	5	33.3	0	0.0	0	0.0	0	0.0	0	0.0	0	0.0
Blood bilirubin increased	3	20.0	0	0.0	0	0.0	0	0.0	0	0.0	0	0.0
Lymphocyte count decreased	1	6.7	1	6.7	0	0.0	0	0.0	0	0.0	0	0.0
Urine output decreased	1	6.7	1	6.7	0	0.0	0	0.0	0	0.0	0	0.0

The most common grade 3 event was anaemia with an incidence of 53.3%. The most common grade 4 events were “platelet count decreased,” “neutrophil count decreased,” and “white blood cell counts” decreased in 40.0%, 33.3%, and 26.7% of patients, respectively. For other grade 4 adverse events, “hypokalaemia” (13.3%), “febrile neutropenia,” “sepsis,” “blood creatinine increased,” and “lymphocyte count decreased” and “hypocalcaemia” (each 6.7%) were observed. The mean AST (GOT) and ALT (GPT) decreased after administration of glucarpidase. No clinically significant changes occurred for any other laboratory values; occult blood was not detected in urine.

The proportion of patients with MTX-related adverse events included in the secondary analysis was 64.3% (9/14 patients). By event, the progression of renal impairment was most frequent in 8 patients, followed by pyrexia in 6 patients, febrileneutropenia in 4, severe mucositis in 2, and infections and infestations in one. The nonoccurrence rate of MTX-related adverse events was 35.7% (5/14 patients).

In the OP-07-001 study, a total of 15 adverse events occurred in all 4 patients (100.0%). The causal relationship to the investigational drug was ruled out except for one case (headache), and most events were attributable to MTX. Pyrexia occurred for 2 patients but the event was not related to glucarpidase.

### Antibody results

In the CPG2-PII study, the proportion of patients with antibodies to glucarpidase before the start of administration of glucarpidase and at 1, 3, and 6 months after this was 13.3%, 33.3%, 13.3%, and 15.4% (2/15, 5/15, 2/15, and 2/13 patients, respectively).

### Pharmacokinetic profiles of glucarpidase in CPG2-PII and OP-07-001 studies

In both trials, the C_max_ was reached approximately 20 min after the administration of glucarpidase, and subsequently decreased over time. The area under the plasma concentration–time curve from 0 to 24 h (AUC_0–24 h_) mean values were 15.1 and 12.9 ng·h/mL, and the mean plasma elimination half-life (t_1/2_) was 5.61 and 5.72 h in CPG2-PII and OP-07-001 studies, respectively (Table 3).

**Table 3:** Glucarpidase pharmacokinetic parameters

PK parameters	CPG2-PII (*N* = 15)	OP-07-001 (*N* = 4)	Reference* (*N* = 8)
C_max_ (ng/mL)	2320 ± 617	1910 ± 610	3080 ± 843
t_max_ (h)	0.35 (0.23–0.43)	0.37 (0.33–0.40)	0.25 (0.10-2.00)
t_1/2_ (h)	5.61 ± 0.759	5.72 ± 0.697	9.00 ± 3.18
AUC_0 − 24 h_ (µg・h/mL)	15.1 ± 3.07	12.9 ± 5.41	NC
AUC_0 − inf_ (µg・h/mL)	15.9 ± 3.40	13.6 ± 5.88	23.4 ± 6.85
CL_tot_ (mL/min/kg)	0.111 ± 0.0228	0.141 ± 0.0506	0.0892 ± 0.0302
V_d,ss_ (mL/kg)	38.2 ± 7.01	62.5 ± 17.5	58.0 ± 18.1

Data are mean ± SD, except for t_max_, which is represented as median and range. NC, not calculation

*This was an open-label, single site study to characterize the pharmacokinetics and assess the safety and tolerability of glucarpidase in subjects who had both normal and severely impaired renal function. In the parameters, values for 8 subjects (6 Black and 2 Caucasian) with normal renal function, as measured by ELISA, were documented [40]

### Pharmacokinetic profiles of DAMPA, LV, and 5-MeTHF in the CPG2-PII study

In the CPG2-PII study, the pharmacokinetic profiles of DAMPA, LV, and 5-MeTHF were analyzed as secondary end points. Each data is shown in Supplementary Table S4. Briefly, the C_max_ (mean ± SD) of DAMPA after the start of administration of glucarpidase was 18,400 ± 46,600 ng/mL, and the median t_max_ (min–max) was 47.22 (26.75–99.67) h. The C_max_ (mean ± SD) of LV after the start of administration of glucarpidase was 27,500 ± 34,000 ng/mL, and the median t_max_ (min–max) was 16.20 (0.72–24.00) h. The C_max_ (mean ± SD) of 5-MeTHF after the start of administration of glucarpidase was 815 ± 761 ng/mL, and the median t_max_ (min–max) was 16.68 (0.00–23.43) h.

## Discussion

The rate of CIR (and two-tailed 95% CI) among the 13 patients included in the primary analysis of the CPG2-PII study was 76.9% (46.2–95.0%) (Fig. 1). In the OP-07-001 study, which was conducted using a measurement method that stopped enzyme activity during blood collection, the plasma MTX concentration was rapidly reduced and maintained at or below the threshold (1 µmol/L) thereafter. The rate of MTX concentration reduction (central measurement) in the OP-07-001 study was similar to that in the CPG2-PII study, showing the robustness of the decreasing rate of plasma MTX concentration 20 min after the glucarpidase administration. These CIR results were similar to those in a US study [28], which used pooled analysis of efficacy data from four multicenter, single-arm, compassionate-use clinical trials using protocols from 1993 to 2007. Efficacy was defined as rapid and sustained CIR (RSCIR) in plasma methotrexate concentration, at 1 µmol/L or lower at all post-glucarpidase determinations. Plasma methotrexate concentrations demonstrated consistent 99% median reduction, and RSCIR was achieved by 83 (59%) of 140 patients.

Our studies showed that glucarpidase rapidly metabolizes the circulating extracellular MTX and thereby reduced plasma MTX concentrations by > 98% within 20 min of administration. In addition, this catalytic effect on circulating MTX persists for approximately 48 h. However, because MTX is also distributed into tissues, redistribution can occur from tissues to the blood following concentration gradients. Therefore, at 48 h, the MTX concentration increased (i.e., rebound phenomenon) (Fig. 2) [29, 30]. Although the plasma level of MTX in rebound has been shown to be substantially lower than the MTX level before treatment with glucarpidase [28] and in our studies, most patients did not exceed the 1 µmol/L threshold, continuous monitoring of MTX concentrations is important to prevent adverse events that rebound MTX may cause. In addition, glucarpidase cannot directly affect the level of intracellular MTX, and only LV can rescue normal cells. Therefore, continuous LV administration is also needed. Rebounds were identified in several patients in the CPG2-PII study; however, 2 patients (Patients #2 and #8) could not achieve CIR due to rebounds.

MTX was measured by LC/MS/MS at a central laboratory in this study, but most institutions use the immunoassay method which does not distinguish between MTX and its metabolite DAMPA. Therefore, MTX measured by immunoassay comprises both MTX and DAMPA, and so these immunoassays do not give reliable measurements for MTX after glucarpidase treatment. In a pooled analysis of 169 patients from two US and two EU single-arm, multicenter, compassionate-use studies, further reductions in the MTX levels were noted in 8 patients with MTX concentrations of > 1 µmol/L after a first dose of glucarpidase and who received a second dose ≥ 48 h later. However, the extent of reductions in the MTX levels varied considerably between these patients, with individual reductions varying from 8 to 97% [28]. Theoretically, MTX amount that the second dose of glucarpidase can degrade becomes less than that the first dose can. Depending on the MTX concentration before the first dose, the second dose of glucarpidase has some clinical significance; however, the second dose of glucarpidase should be considered carefully, accounting the costs of drugs. The glucarpidase-MTX popPK analysis and Bayesian estimation of rebound in plasma MTX concentrations (which was reported previously) may support early decision-making for the second dose of glucarpidase [31].

The LV dose should be adjusted depending on the MTX concentration but this should be decided depending on the MTX concentration at baseline before glucarpidase administration because MTX concentrations that are measured by immunoassay method include DAMPA. Furthermore, LV rescue therapy should be continued at least 48 h after administration of glucarpidase [32].

Serum creatinine was known to be correlated with MTX concentration [33]. The transitions of the MTX concentration and serum creatinine level after HD-MTX have been described in a case report [34]. A high level of MTX concentration at 48 h after administration of MTX was recognized in case 1 and 2 patients; subsequently, the serum creatinine level rapidly increased late and decreased after several days. In case 3 patient, the MTX concentration after HD-MTX was similar to that in case 2 patient; however, a rapidly increasing serum creatinine level was not noted in case 3 patient. It was considered that the MTX concentration after HD-MTX in case 3 patient was lower than those in case 1 and 2 patients and decreasing speed in MTX concentration was early after 48 h from administration of MTX. From the aforementioned reason, a rapid decrease in the MTX concentration is considered important. The serum creatinine levels in the CPG2-PII study increased shortly after administration of glucarpidase and then decreased to baseline as the study progressed, as demonstrated in the results shown in Fig. 3a and b. The transition of the serum creatinine level in most of patients was similar to that in case 3 patient [34]. This may have occurred because administration of glucarpidase led to a rapid decrease in the MTX concentration. Patient 1 showed an increased serum creatinine level after administration of glucarpidase, probably because this patient had acute renal failure and the baseline MTX concentration exceeded 400 µmol/L. In patient 3, receiving cimetidine, which causes serum creatinine increments after glucarpidase, led to the possibility that serum creatinine in this patient increased dramatically [35]. Therefore, glucarpidase may be useful for the management and prevention of HD-MTX-induced toxicity, especially kidney injury.

Most adverse events were determined to be MTX-related, whereas 35.7% (5/14) of the patients experienced no MTX-related adverse events in the CPG2-PII study. Although high-dose LV therapy in clinical practice is useful for the reduction in MTX-related adverse events, most cases develop such adverse events with this therapy [36]. Thus, glucarpidase may reduce adverse events that occur in case of delayed MTX clearance.

The adverse events of hypersensitivity and hypertension that occurred in the CPG2-PII study and are listed as significant adverse events in the overseas approval application were investigated. Two events of “hypersensitivity” occurred in one patient. Seven sites of a bulging rash appeared on the head approximately 30 min after administration of glucarpidase in 1 patient and disappeared approximately 1 h later without treatment. Although antibodies to glucarpidase were detected in several patients 1, 3, and 6 months after administration of the glucarpidase, those antibodies did not affect the activity of glucarpidase in lowering the MTX level. Although the number of patients in this study was not sufficient to reach firm conclusions, the production of anti-glucarpidase antibodies reported for several patients in the CPG2-PII study suggests that caution is required regarding hypersensitivity. These safety results were similar to those in the previous report [26].

Therefore, the results reported in this manuscript showed that one or two administrations of glucarpidase 50 U/kg in Japanese patients with a MTX clearance disorder were highly effective in lowering the plasma MTX concentration with a high level of safety and favorable tolerability of glucarpidase. The pharmacokinetic parameters of glucarpidase in both studies were similar to those in the previous report [26]. Since the half-life of glucarpidase is short, glucarpidase cannot metabolize MTX migrating from tissues into blood vessels and LV rescue therapy should be continued until below the specified concentration. Furthermore, LV should not be given concomitantly with glucarpidase or within 2 h as it could reduce glucarpidase efficacy [37]. As similar clinical benefits were suggested in Japanese patients, based on data, including the results of these trials, glucarpidase was eventually approved in Japan in 2021.

LV is administered after glucarpidase treatment because of MTX reentering the plasma from the tissue. However, HD-MTX failures have been reported in association with higher LV doses in pediatric patients [24]. A high LV dose is related to a higher risk for relapse and doubling of the dose increased the relapse risk by 22% [25]. Leucovorin has optical isomers, D,L-5-formyltetrahydrofolate. Glucarpidase selectively degrades L-5-formyltetrahydrofolate [38] and elimination half-life is reported as 32–35 min [39].

Excessive LV rescue therapy could thus interfere with the MTX efficacy. Glucarpidase can effectively reduce MTX levels in blood, and administering glucarpidase instead of an excessive amount of LV to patients with MTX toxicity may help avoid the risk of recurrence.

In conclusion, one or two doses of glucarpidase at 50 U/kg in Japanese patients with a MTX clearance disorder were effective in reducing the plasma MTX concentration and may mitigate MTX-related adverse events, and favorable safety and tolerability of glucarpidase were confirmed.

### Electronic supplementary material

Below is the link to the electronic supplementary material.

Supplementary Material 1

## Data Availability

The datasets used and/or analysed during the current study are available from the corresponding author on reasonable request.
