# Supplementary material for: Phase 2 study of glucarpidase in patients with delayed methotrexate elimination after high-dose methotrexate therapy
Source: Cancer Chemother Pharmacol. 2024 Mar 13;94(1):89–101. doi: 10.1007/s00280-024-04664-6 (PMC11258064; doi:10.1007/s00280-024-04664-6)
Supplement: Supplementary file 1 — Supplementary Material 1 [file 280_2024_4664_MOESM1_ESM.docx]

**Phase 2 study of glucarpidase in patients with delayed methotrexate elimination after high-dose methotrexate therapy**

**Journal name:** Cancer Chemotherapy and Pharmacology

Atsushi Ogawa^1*^, Hiroshi Kawamoto^2^, Junichi Hara^3^, Atsushi Kikuta^4^, Chitose Ogawa^5^, Hiroaki Hiraga^6^, Kenichi Yoshimura^7^, Kazunari Miyairi^8^, Reiko Omori^8^, Tokihiro Ro^8^, Yuna Kamei^8^ and Toshimi Kimura^9^

^1^ Department of Pediatrics, Niigata Cancer Center Hospital, Niigata, Japan

^2^ Department of Pediatric and Allergy, Fujimi Clinic, Tokyo, Japan

^3^ Department of Pediatrics Hematology/Oncology, Osaka City General Hospital, Osaka, Japan

^4^ Department of Pediatric Oncology, Fukushima Medical University Hospital, Fukushima, Japan

^5^ Department of Pediatric Oncology, National Cancer Center Hospital, Tokyo, Japan

^6^ Department of Musculoskeletal Oncology, NHO Hokkaido Cancer Center, Sapporo, Japan

^7^ Department of Biostatistics and Data Science, Graduate School of Medical Science Nagoya City University, Nagoya, Japan

^8^ Ohara Pharmaceutical Co., Ltd., Tokyo, Japan

^9^ Department of Pharmacy, Juntendo University Hospital, Tokyo, Japan

***Corresponding author:**

Atsushi Ogawa, MD

Department of Pediatrics, Niigata Cancer Center Hospital

2-15-3 Kawagishi-cho Chuo-ku, Niigata, 951-8566, Japan

E-mail: atsushi@niigata-cc.jp

Tel: 81 25 266 5111, Fax: 81 25 233 3849

**Supplementary Table S1:** Inclusion criteria

**CPG2-PII study**

Inclusion criteria for subjects in the study were as follow.

1. MTX has not been administered, excluding cerebrospinal injection, after administration of MTX at 1 g or more per m^2^ body surface area per dose, and 15 h or longer have elapsed since the end of administration of MTX. It does not matter how long it takes to administer the MTX.
2. a or b of the following criteria is met:
3. No previous use of glucarpidase and either of the following criteria i) to vii) is met.
4. Blood MTX levels of ≥50 μmol/L at least 22 h after the start of MTX administration
5. Blood MTX levels of ≥5 μmol/L at least 40 h after the start of MTX administration
6. Blood MTX levels of ≥2 μmol/L at least 46 h after the start of MTX administration
7. MTX blood concentration ≥1 µmol/L, and signs of acute renal failure at 40 h or later after the start of MTX*
8. MTX blood concentration ≥0.4 µmol/L, and signs of acute renal failure at 46 h or later after the start of MTX*
9. MTX blood concentration ≥0.1 µmol/L (with a dose of MTX 1–3.5 g/m^2^) at 70 h or later after the start of MTX
10. MTX blood concentration ≥0.3 µmol/L (with a dose of MTX >3.5 g/m^2^) at 70 h or later after the start of MTX
11. Previous use of glucarpidase, and signs of acute renal failure, and MTX blood concentration ≥50 µmol/L at 22 h or later after the start of MTX

* A sign of acute renal failure is either of the following.

1. Levels of serum creatinine exceeding the upper limits specified in the following table, at not less than 12 h after the start of MTX administration, or a creatinine clearance or glomerular filtration rate (calculated value or actual measurement, for both) of <70 mL/min.
2. Levels of serum creatinine showing more than a twofold increase from before MTX administration or showing more than a 1.5-fold increase at the last two sequential blood samplings, as well as continuing to increase.

| **Age** | **Upper limit of creatinine** | |
| --- | --- | --- |
|  | **Male** | **Female** |
| Older than 1 year, younger than 2 years | 0.6 | 0.6 |
| Older than 2 years, younger than 6 years | 0.8 | 0.8 |
| Older than 6 years, younger than 10 years | 0.9 | 0.9 |
| Older than 10 years, younger than 13 years | 1.1 | 1 |
| Older than 13 years, younger than 16 years | 1.5 | 1.2 |
| Older than 16 years | 1.7 | 1.2 |

1. Written consent to participate in this study was obtained from the subject or his/her legally authorized representative*.

* The legally authorized representative shall be the subject’s parent, spouse, guardian, or a person equivalent to these, who will act in the best interest of the subject from the viewpoint of their real life and mental cooperative relationship. However, when it is difficult/impossible for the subject to give written consent as an act due to time/geographical limitation, he/she shall be deemed to meet the criterion (3) if all following conditions are satisfied. The verbal confirmation under (iii) shall be recorded in the source document and signed consent form.

1. An explanation about the study has been provided in advance.
2. Written consent has been obtained for study participation when the eligibility criteria are met.
3. Consent of the subject’s representative has been confirmed verbally when the subject meets the other eligibility criteria than (3) and none of the exclusion criteria.

**OP-07-001 study**

(1) Patients whose written consent to participate in this study has been obtained from the patient or his/her legally authorized representative

(2) Patients who have passed at least 15 h after the end of MTX treatment and meet any of the following criteria 1)–4)

1) The blood MTX concentration measured at the site is >50 μmol/L at 22 h or more after the start of MTX administration

2) The blood MTX concentration measured at the site is >5 μmol/L at 40 h or more after the MTX administration

3) The blood MTX concentration measured at the site is >2 μmol/L at 46 h or more after the MTX administration

4) The blood MTX concentration measured at the site is >1 μmol/L at 40 h or more after the start of MTX treatment, and a sign of acute kidney injury^*^ is observed.

*A sign of acute kidney injury is defined as meeting either of the following criteria, (i), (ii), and (iii):

(i) Serum creatinine level after the MTX administration is higher than the upper limit of the site reference value (refer to “Pocket Guide for Reference Laboratory Values in Children [Toshiaki Tanaka, Jiho, 2^nd^ edition]” if no pediatric reference laboratory value is defined)

(ii) At least a 1.5-fold increase in the serum creatinine level from baseline after the MTX administration

(iii) Increase in serum creatinine level by 0.3 mg/dL or higher within 48 h

**Supplementary Table S2:** Blood sampling points in the CPG2-PII study

| **Point**  **Item** | **Day 1** | | | | |
| --- | --- | --- | --- | --- | --- |
|  | **1^st^** | **2^nd^** | **3^rd^** | **4^th^** | **5^th∗1^** |
|  | **0 (just before glucarpidase dosing)** | **20 min after glucarpidase dosing** | **2 h after glucarpidase dosing (just before LV dosing)** | **3 h after glucarpidase dosing** | **5–8 h after glucarpidase dosing (just before the second LV dosing)** |
| glucarpidase | 0 | 20 min | 2 h | ‒ | 5–8 h |
| MTX | 0 | 20 min | 2 h | ‒ | 5–8 h |
| DAMPA | 0 | 20 min | 2 h | ‒ | 5–8 h |
| LV | ‒ | ‒ | 0 | 1 h | 3–6 h |
| 5-MeTHF | ‒ | ‒ | 0 | 1 h | 3–6 h |

| **Point**  **Item** | **Day 2** | | | **Day 3** | **Day 4** | **Day 5** |
| --- | --- | --- | --- | --- | --- | --- |
|  | **1^st^** | **2^nd^** | **3^rd^** | **1^st^** | **1^st^** | **1^st^** |
|  | **14‒20 h after glucarpidase dosing (just before LV dosing)** | **15‒21 h after glucarpidase dosing (1 h after LV dosing)** | **20‒26 h after glucarpidase dosing (just before the next LV dosing)** | **48 h post-glucarpidase dose** | **72 h after glucarpidase dosing** | **96 h after glucarpidase dosing** |
| glucarpidase | ∗^2^ | ‒ | ∗^2^ | 48 h | ‒ | ‒ |
| MTX | ‒ | ‒ | ∗^2^ | 48 h | 72 h | 96 h |
| DAMPA | ‒ | ‒ | ∗^2^ | ‒ | ‒ | ‒ |
| LV | 0 | 1 h | 3–6 h | ‒ | ‒ | ‒ |
| 5-MeTHF | 0 | 1 h | 3–6 h | ‒ | ‒ | ‒ |

∗1: Based on the half-life of LV, the 5^th^ sampling on Day 1 and the 3^rd^ sampling on Day 2 should be conducted at least 6 h after administration of LV. However, in the study, sampling was conducted just before the next LV administration because the interval of dosing LV ranged from 3 to 6 h depending on the blood MTX concentration and differed depending on the LV rescue regimen.

∗2: The blood sampling points on Day 2 varied according to the glucarpidase administration time.

**Supplementary Table S3:** Patients’ demographics and reduction of MTX level

| **Study no.** | **Patient #** | **Demographics** | | | | **Creatine  at Baseline^#^ (mg/dL)** | **MTX Concentration  (µmol/L)** | | **Reduction Rate at 20 min  (%)** | **CIR  achieved** |
| --- | --- | --- | --- | --- | --- | --- | --- | --- | --- | --- |
|  |  | **Age (years)** | **Gender** | **Body weight (kg)** | **Diagnosis^§^** |  | **Baseline** | **20 min^##^** |  |  |
| CPG2-PII | 01 | 14 | Female | 50 | OS | 2.71 | 486.093 | 5.184 | 98.9 | no |
| CPG2-PII | 02 | 16 | Male | 55.4 | ALL | 1.68 | 22.819 | 0.209 | 99.1 | no |
| CPG2-PII | 03 | 14 | Female | 52.3 | OS | 0.81 | 45.683 | 0.585 | 98.7 | yes |
| CPG2-PII | 04 | 12 | Male | 34.4 | OS | 0.67 | 26.098 | 0.230 | 99.1 | yes |
| CPG2-PII | 05^*^ | 12 | Male | 33.2 | OS | 0.69 | 77.194 | 0.486 | 99.4 | - |
| CPG2-PII | 06 | 16 | Male | 47 | OS | 0.78 | 75.808 | 0.492 | 99.4 | yes |
| CPG2-PII | 07 | 47 | Male | 65.8 | OS | 1.01 | 23.215 | 0.143 | 99.4 | yes |
| CPG2-PII | 08 | 15 | Female | 43 | ALL | 1.25 | 26.890 | 0.364 | 98.6 | no |
| CPG2-PII | 09 | 27 | Male | 76 | OS | 1.47 | 41.084 | 0.349 | 99.2 | yes |
| CPG2-PII | 10^**^ | 1 | Female | 12.1 | NHL | 0.23 | 0.680 | 0.008 | 98.8 | - |
| CPG2-PII | 11 | 1 | Male | 10.7 | CM | 0.42 | 2.988 | 0.018 | 99.4 | yes |
| CPG2-PII | 12 | 17 | Male | 39.8 | OS | 0.7 | 1.814 | 0.016 | 99.1 | yes |
| CPG2-PII | 13 | 8 | Female | 22.1 | NHL | 0.75 | 7.022 | 0.068 | 99.0 | yes |
| CPG2-PII | 14 | 33 | Male | 78.1 | ALL | 1.8 | 6.161 | 0.074 | 98.8 | yes |
| CPG2-PII | 15 | 75 | Female | 66.1 | OS | 3.47 | 54.177 | 0.388 | 99.3 | yes |
| OP-07-001 | OP07-01-01 | 2 | Male | 12.6 | ALL | 0.22 | 1.483 | 0.019 | 98.7 | yes |
| OP-07-001 | OP07-01-02 | 79 | Male | 62.2 | NHL | 3.72 | 2.746 | 0.022 | 99.2 | yes |
| OP-07-001 | OP07-02-01 | 2 | Female | 12.6 | ALL | 0.22 | 1.810 | 0.026 | 98.5 | yes |
| OP-07-001 | OP07-02-02 | 17 | Male | 55.1 | ALL | 0.72 | 2.073 | 0.023 | 98.9 | yes |

*: Excluded from the efficacy analysis due to secondary entry of patient #04

**: Excluded from the efficacy analysis due to low MTX Level at Baseline (below 1 µmol/L)

§: Abbreviations of Diagnosis

ALL: Acute Lymphoblastic Leukemia OS: Osteosarcoma NHL: Non-Hodgkin’s Lymphoma CM: Cerebellar Medulloblastoma

#: At the time of glucarpidase administration

##: At 20 min after glucarpidase administration

**Supplementary Table S4:** Pharmacokinetic parameters in CPG2-PII study

| **PK parameters** | **CPG2-PII** | | | | | | | | |
| --- | --- | --- | --- | --- | --- | --- | --- | --- | --- |
|  | **DAMPA (N = 15)** | | | **LV (N = 15)** | | | **5-MeTHF (N = 15)** | | |
| C_max_ (ng/mL) | 18400 | ± | 46600 | 27500 | ± | 34000 | 815 | ± | 761 |
| t_max_ (h) | 47.22 | (26.75–99.67) | | 16.20 | (0.72–24.00) | | 16.68 | (0.00–23.43) | |
| t_1/2_ (h) | 19.4 | ± | 6.56 | 65.7 | ± | 39.6^a^ | 37.4 | ± | 26.4^a^ |
| AUC_0–24h_ (µg・h/mL） | 120 | ± | 271 | 326 | ± | 168^b^ | 12.7 | ± | 12.4^b^ |
| AUC_0-t_ (µg・h/mL） | 534 | ± | 1580 | 242 | ± | 193 | 8.11 | ± | 8.60 |
| AUC_0-inf_ (µg・h/mL） | 537 | ± | 1590 | 1050 | ± | 323^a^ | 38.4 | ± | 16.9^a^ |

Data are presented as mean ± SD, except for t_max_, which is given as median and range.

a; n = 2

b; n = 3
